# World Health Organization African Region national heads of units of diagnostics and laboratory services meetings proceedings

**DOI:** 10.1186/s12919-024-00305-1

**Published:** 2024-11-28

**Authors:** Sheick Oumar Coulibaly, Michelle Amri, Christine Vuguziga, Aminata Binetou-Wahebine Seydi, Lorna Maria Aine, Bertha Kembabazi, Sokona Sy, Lindiwe Elizabeth Makubalo

**Affiliations:** 1 WHO Regional Office for Africa, Office of the Assistant Regional Director, Cité du Djoué, Brazzaville, PB06 Republic of the Congo; 2 The W. Maurice Young Centre for Applied Ethics, School of Population and Public Health, University of British Columbia, 2206 East Mall, Vancouver, BC V6T 1Z3 Canada

**Keywords:** Diagnostic and laboratory services, Primary health care, Health systems, Global health, Health governance, Multisectoral action, Public policy, Public administration, Universal Health Coverage, Sustainable Development Goals, Africa, World Health Organization

## Abstract

**Background:**

In the World Health Organization (WHO) African Region, many cases of serious and preventable diseases remain unmanaged because appropriate and good quality diagnostic support is not available at all levels within countries’ health systems. Diagnostic and laboratory services influence the efficiency and effectiveness of both clinical and public health functions, including diagnosis, treatment, health promotion, disease prevention, surveillance and response, and research. Essential to global health security, these services are vital to decision-making processes by clinicians, epidemiologists, public health specialists, and health policymakers. To update, promote, and reinforce diagnostic and laboratory services, it was deemed necessary to organize consultation meetings. These consultation meetings hosted: national diagnostic and laboratory directors or heads of units within ministries of health; officers in charge of laboratories from WHO country offices; representatives of the WHO African Regional Office (AFRO) clusters, units, and Headquarters; experts; and strategic partners. This article details the consultation meetings hosted in Lomé, Togo from 14 to 17 June 2022 and in Kigali, Rwanda from 5 to 7 July 2022.

**Methods:**

Although the two meetings were made distinct due to their different operating languages—French and English, respectively—each consultative meeting sought to engage participants in the same thematic areas of discussion, thus containing the same presentations and areas of discussion. This article compiles the presentations of both meetings, where a total of 85 individuals attended, reflecting 30 countries in the African Region.

**Results:**

Summaries of technical presentations at both meetings are provided, which have the following titles: (1) “AFRO: new vision for the laboratory sector;” (2) “WHO strategies for strengthening laboratories;” (3) “Collaborative registration process for in vitro diagnostic products: introduction and implementation;” (4) “Status of diagnostics and laboratory regulations in the African Region;” (5) “Health technology management;” (6) "The Global Laboratory Leadership Programme;” (7) “Model list of essential in vitro diagnostic devices;” (8) “Primary health care & laboratory and diagnostic services;” (9) “Antimicrobial resistance control and laboratory systems;” and (10) “Integrated laboratory systems’ contributions to disease control programmes.”

**Discussion:**

Following the technical presentations, thematic exchanges were planned around six key areas, with one country presenting their experiences per theme, both at the meeting held in French and for the meeting held in English. Therefore, two countries’ experiences are detailed around each of the six thematic areas, which are: laboratory governance (Guinea and Sierra Leone), laboratory policy and planning (Togo and Zimbabwe), regulation and legislation (Senegal and Ghana), partnerships (the Democratic Republic of the Congo and Nigeria), management of laboratory technology (Gabon and Zambia), and national laboratory networks (Burkina Faso and Rwanda).

**Conclusion:**

Through these meetings, laboratory leaders were able to not only learn from best practices and anticipate challenges for their own respective countries, but also benefit from joining a platform for laboratory leaders to foster cross-country connections for the duration of their careers. Ultimately, these meetings signal the beginning of many fruitful collaborations and opportunities in the African Region in laboratory and diagnostic services.

**Supplementary Information:**

The online version contains supplementary material available at 10.1186/s12919-024-00305-1.

## Background

Laboratory services influence the efficiency and effectiveness of clinical and public health functions — including diagnosis, treatment, health promotion, disease prevention, surveillance and intervention, and research. Laboratory services are indispensable for primary health care (PHC), as they are essential to guide the decision-making processes of clinicians, epidemiologists, public health specialists, and health policymakers, and for global health security more generally [1, 2].

Given that universal health coverage (UHC) is the focus of the World Health Organization’s (WHO) thirteenth general programme of work (GPW 13), along with ongoing UHC-focused efforts of member state countries in the Region, it was critical for the Regional Office of Africa (AFRO) to take stock of policies, strategies, plans, and activities in relation to diagnostics and laboratories and share updates from the WHO. Thus, it was deemed necessary to organize consultation meetings hosting national diagnostic and laboratory directors; officers of WHO country offices in charge of laboratories; representatives of AFRO clusters, units, and Headquarters; experts; and strategic partners. This article details the proceedings of these consultation meetings hosted in Lomé, Togo from 14 to 17 June 2022 and in Kigali, Rwanda from 5 to 7 July 2022. Although the two meetings were made distinct due to their different operating languages — French and English, respectively — both consultative meetings sought to engage participants in the same areas of discussion. As such, this article compiles the proceedings of both meetings.

## Methods

For the Lomé meeting, the first day provided the opportunity for presenters to share their knowledge on the new WHO vision, strategies on laboratories, and different topics for which participants presented observations, questions, concerns, and advice. The second and third days allowed participants to share their respective experiences and engage in thematic exchanges on laboratory and diagnostic services.

For the Kigali meeting, the first day allowed presenters to share their knowledge on the new WHO AFRO vision for the laboratory sector, WHO strategies for strengthening laboratories, and other technical presentations. The second and third days provided an opportunity for participants to exchange experiences from their respective countries across six thematic areas around laboratory and diagnostic services.

Both meetings closed by reiterating the desire of the WHO to host this event to create a platform of laboratory leaders to foster connections and allow for experience-sharing. Across both meetings, 85 individuals attended, reflecting 30 countries in the African Region (for a full list of participants, please see the supplemental file).

## Technical presentations

### AFRO: new vision for the laboratory sector

Dr. Sheick Oumar Coulibaly, the team lead for Diagnostics and Laboratory Services at WHO AFRO, briefly discussed the ongoing challenges faced in the Region, including challenges resulting from the COVID-19 pandemic. To overcome these challenges, the adoption of the WHO’s GPW 13 — where the three main strategic objectives are UHC, health and well-being, and health emergencies — will be used to guide the new vision for the laboratory sector. To introduce the topic, Dr. Coulibaly noted that laboratory services receive little or no attention in many countries in the African Region. As a result, there is a lack of priority in terms of funding, planning, and service delivery, hence the need to strengthen health laboratories. Dr. Coulibaly noted that evidently not all countries in the world have the same systems in place, drawing on the finding from *The Lancet* Commission on diagnostics noting that 47% of the world's population has little or no access to diagnostics [3], and observed that this figure is likely higher in Africa.

Dr. Coulibaly provided the imagery of a chariot following behind cattle that leads to results and juxtaposed this with cattle following behind the chariot, to provide an analogy that, without laboratory services, treatment is blind. This analogy was drawn on to emphasize that time and money are not lost by going to the laboratory for a test, as such actions can help manage cases more rationally and efficiently. This also led to a brief reminder on the scope of laboratory services, indicating that laboratory services serve many areas in the health sector, including general clinical medicine, epidemic detection, disease surveillance, research, and many more areas, thus the focus of actions should not be narrow.

Dr. Coulibaly reiterated that WHO AFRO’s new vision is based on PHC, the integration of diagnostics including laboratory activities, and the diversification of WHO actions. First, for PHC, this entails reorganizing the structure of the Regional Office; promoting diagnostics and laboratory efforts following the GPW 13; creating a diagnostics and laboratory unit; integrating and coordinating efforts; developing a regional strategy; prioritizing the diagnostics and laboratory in dialogues with Member States; harmonizing units in charge of PHC, health products, and health systems; and bringing services closer to countries (e.g. through multi-country assignment teams). Second, in integrating laboratory activities, this entails expanding the diagnostics sector to include all technical areas of WHO AFRO’s activities (e.g., regulation, maintenance, service delivery); better coordinating diagnostics activities through clusters, programmes, and areas of work; “bottom-up” common planning with clusters and programmes; and pooling and better utilizing resources. And lastly, in diversifying the WHO’s actions, this entails having greater consideration and involvement in the areas of diagnostics legislation and regulation; enhancing support to Member States to improve governance and structuring of the diagnostics sector; developing diagnostics leadership; better considering and developing diagnostics and laboratory equipment management; and envisioning diagnostics services at health care facilities as a full part of service delivery. Dr. Coulibaly concluded his session by referencing a photo from Benin that illustrates Beninese wisdom and states that when several fingers are put together, they can plug the holes of a canary, which is a traditional water storage container, and thus preserve the water in it from running out, to emphasize that together we are stronger.

### WHO strategies for strengthening laboratories

This presentation was conducted by two different speakers. The synopsis of the presentation in French by Dr. Sébastien Cognat is described first, followed by the synopsis of the presentation in English by Dr. Céline Barnadas.

Dr. Sébastien Cognat, the Head of the Public Health Laboratory Strengthening unit in the Preparedness Division for the Health Emergencies Programme at the World Health Organization Headquarters, discussed state of the art laboratory services, noting four key themes indicating the challenges faced by these services, WHO strategies to strengthen laboratory services, and key considerations. Dr. Cognat observed that there has not yet been a unified strategy for strengthening laboratory services in Africa.

Dr. Cognat presented challenges related to access to quality tests; organized networks from the community to the tertiary level; reimbursement by health insurance systems; capacity for research and development, production, validation, and distribution of tests; retention of skilled human resources; and vertical models of funding and assistance. Dr. Cognat also provided recommendations for laboratory strengthening, which included: well-governed laboratory networks, guaranteed access to innovative and quality tests, highly competent and empowered laboratory personnel, timely sharing of laboratory data for public health measures, and laboratory preparedness for priority risks. Lastly, Dr. Cognat presented a six-pillar strategy: policies, strategies, and plans; national diagnostic capacity; quality systems; workforce development; networks and coordination; and biosafety and biosecurity.

For the presentation in English, Dr. Céline Barnadas, a Technical Officer in the Public Health Laboratory Strengthening team from Lyon, France, began this session by reiterating the views of Dr. Coulibaly around the importance of diagnostic tests. Dr. Barnadas expressed that there is global recognition of the need to strengthen diagnostics and laboratory services from the community to the global level, which is noted in the Rome Declaration of 2021 [4], along with emergency committee meetings of the International Health Regulations [5, 6], The Independent Panel for Pandemic Preparedness & Response’s main report [7], and in the World Health Assembly’s Resolution 74.7 [8]. Dr. Barnadas further noted that WHO strategies to strengthen laboratories are often focused on specific diseases or programmes, particularly during the COVID-19 pandemic, which also aligns with the tendency for funding to be allocated to vertical programmes.

Dr. Barnadas indicated that there are five critical elements for strengthening laboratories:

1. Well-governed and cost-effective public health laboratory networks
  The WHO has been working to establish and strengthen multisectoral national public health laboratory working groups; develop and revise national public health laboratory emergency preparedness policies, strategies, and regulatory frameworks; and design tiered national public health laboratory networks.

2. Access ensured to innovative and quality-assured testing
  This entails enhancing integrated specimen collection and transport systems; promoting research, development of, and access to new and innovative technologies (e.g., sequencing); capacitating the laboratory network with fit-for-purpose quality-assured diagnostics and integrated platforms cutting across diseases and safe, secure, and properly maintained infrastructure and equipment; and ensuring quality compliance with internationally recognized quality standards towards accreditation.

3. A highly competent and empowered laboratory workforce
  One of the key areas under this element is to foster a new cadre of laboratory leaders and managers. As well, increasing staff retention through continuous training opportunities and improved career pathways, and promoting knowledge-sharing through communities of practice.

4. Laboratory data shared in a timely manner for public health action
  This entails generating quality data through inter-operable Laboratory Management Information Systems, sharing data across laboratory networks according to agreed upon standards and principles, and using laboratory data for public health action through connectivity with surveillance and health information systems.

5. Readiness for priority hazards
  This can be improved through identifying public health laboratory network needs and gaps for priority hazards through standardized checklists, action reviews, and simulation exercises; ensuring testing and referral capacity for priority hazards through focused training, procurement, and participation in international networks; and recognizing reference laboratory readiness for specific hazards through a WHO standardized process.

Dr. Barnadas provided examples of two Regions that undertook actions to strengthen laboratory capacity, namely, the WHO Eastern Mediterranean Region, which developed the *Strategic framework for strengthening health laboratory services 2016–2023* [9], and the WHO European Region, which launched *Better Labs for Better Health* [10] that has been ongoing for ten years. With respect to the latter, this entails actions in four key areas: strengthening national laboratory systems, strengthening training programmes and national quality systems, establishing networks for emergency preparedness and response, and advocating for establishing leadership for change and partnerships. Dr. Barnadas concluded by thanking the participants and emphasizing the importance of raising challenges so that the WHO can better support Member States.

### Collaborative registration process for in vitro diagnostic products: introduction and implementation

The presentation in French was conducted jointly by Drs. Aïssatou Sougou and Sunday Kisoma, whereas the presentation in English was provided solely by Dr. Sunday Kisoma. The synopsis for this latter presentation is detailed below.

Dr. Sunday Kisoma, a Consultant for WHO Headquarters within the regulation and qualification department, presented on the collaborative registration process (CRP) for in vitro diagnostic (IVD) products. Dr. Kisoma indicated that CRPs, which began with medicines and moved into IVDs, facilitate the exchange of information to accelerate national registration by providing national regulatory authorities (NRAs) with detailed assessment and inspection reports generated by reference NRAs/prequalification (PQ). Thus, the WHO assists Member States in achieving and maintaining regulatory oversight to build country capacity with good regulatory practices through their PQ CRP. The WHO PQ CRP applies to products prequalified by the WHO, namely, medicines, vaccines, biotherapeutics, and IVDs. The full list of PQ IVDs is periodically updated and is available on the WHO website [11].

As of 30 March 2022, there are 20 participating NRAs. There are five stages for participating in PQ IVDs CRP: (i) NRA agreement, (ii) collaborative registration and submission of application to the NRA, (iii) collaborative registration and access to WHO reports, (iv) regulatory approval, and (v) post-approval changes and monthly updates. After participation in the programme is confirmed, there are various capacity-building opportunities for NRA staff in the CRP programme. In concluding, Dr. Kisoma encouraged meeting participants who are not currently engaged to partake in further discussions.

### Status of diagnostics and laboratory regulations in the African Region

Dr. Edith Andrews Annan, a Technical Officer of medicines and health technologies, presented on the status of regulation of medical devices (MD) including IVDs and laboratory items. Dr. Annan introduced the presentation by emphasizing how critically important the topic is for health care delivery, particularly for safety and efficacy in public health and patient care. Naturally, MDs are wide-ranging in nature, including surgical instruments, medical equipment, IVDs, implantable medical devices, and more, and are used for prevention, diagnosis, and treatment. However, in the African Region, MD regulatory processes are not always well-defined, and many countries rely on clearance from the European Medicines Agency or the United States of America Food and Drug Administration. Notably, in the WHO African Region, 40% of countries have no regulations for MDs, 32% have some regulations, the remaining 28% have no available data, and 60% do not have national lists of essential MDs fulfilling WHO recommendations [12].

The goal of laboratory regulation is to ensure improved access to safe, high quality, affordable, and appropriate MDs, which can be sought through targeting research and development, assessments by ministries of health, regulations by NRAs, and management by ministries of health and health care facilities. Striving for this goal can help move the needle on target 3.8 of the Sustainable Development Goals (SDGs) — achieving UHC through striving to achieve the 255 sub-indicators — as laboratory services are essential to guide decision-making processes by clinicians, epidemiologists, public health specialists, and health policymakers. At present, there are numerous opportunities for regulation by NRAs, which include the opportunity to secure political commitment following the COVID-19 pandemic.

Dr. Annan also provided participants with an update on the WHO Global Benchmarking Tool + Medical Devices (GBT + MD) [13], which allows for evaluation and benchmarking of national regulatory systems of MDs, including IVDs. The GBT + MD revision version one published in April of 2022 is the latest release, which comprises seven regulatory functions under the overarching framework of the national regulatory system.

Dr. Annan concluded by expressing that strong MD regulation is important and needed for achieving higher quality and affordable health care for countries working within tight fiscal constraints. Ultimately, important shifts to achieve leadership centred on vision, anticipation, partnership, and accountability are needed, and harmonization, convergence, and joint efforts need to be promoted.

### Health technology management

Dr. Adriana Velázquez, the Team Lead of the Medical Devices and In Vitro Diagnostics Unit at WHO Headquarters, presented on health technology management virtually for those in Lomé. Dr. Velázquez noted that medical devices are essential for health services, then presented three dimensions of universal coverage and explained the process of medical device needs assessment, as well as their standardization and normalization. Dr. Velázquez also oriented participants to the technical information available at the webinars.

### The Global Laboratory Leadership Programme (GLLP)

This presentation was conducted by two different speakers. The synopsis for the presentation in French by Dr. Virginie Dolmazon is presented first, followed by the synopsis for the presentation in English by Dr. Lisa Stevens.

Dr. Lisa Stevens of the public health laboratory strengthening unit of WHO Headquarters office in Lyon, France presented on the Global Laboratory Leadership Programme (GLLP). Dr. Stevens began the presentation by providing an overview of the goal of the GLLP — to foster and mentor current and emerging laboratory leaders to build, strengthen, and sustain national laboratory systems — and its partners — a multisectoral collaboration of six founding organizations: the Association of Public Health Laboratories, the Centers for Disease Control and Prevention, the European Centre for Disease Prevention and Control, the Food and Agriculture Organization of the United Nations, the World Organisation for Animal Health, and the World Health Organization.

Dr. Stevens further explained how the Laboratory Leadership Competency Framework [14] defines the competencies within the scope of the programme (i.e., what competencies leaders need to have), which fall into nine buckets: laboratory system; leadership; management; communication; quality management system; biosafety and biosecurity; disease surveillance and outbreak investigation; emergency preparedness, response, and recovery; and research. The competency framework can be used by both organizations, providing a foundation for laboratory leadership learning programmes, standardization for workforce development, and others, and by individuals to assess knowledge, skills, abilities, areas for improvement, and plan for achieving higher levels of proficiency.

The GLLP’s learning package contains 43 modules, a planning and implementation guide and toolbox, mentorship guide, and project guide. However, the core GLLP contains didactic sessions of 100 contact hours and mandatory modules equating to 50 contact hours, mentorship, two assignments, and a capstone project. Overall, across the four key areas — training content, mentorship, projects, and community-building — engagement should last at least 24 months.

As of July 2022, the GLLP has engaged 175 total participants, including many participants in the WHO African Region. The GLLP also has a sub-regional programme for Central Africa, which targets five African countries: the Central African Republic, Chad, the Democratic Republic of the Congo, Gabon, and the Republic of the Congo. Dr. Stevens thanked participants and provided relevant email addresses and a weblink to further engage.

Dr. Virginie Dolmazon, from the Public Health Laboratory Strengthening unit in Lyon, France, presented in French on the GLLP in Lomé. Dr. Dolmazon noted that the partnership involves a multisectoral collaboration of six major organizations targeting human and animal health laboratories and laboratories with public health functions (e.g., environmental, agricultural, food, etc.). To provide laboratory professionals with the tools to develop their leadership skills and to invest in and strengthen national laboratory systems with a One Health approach — which recognizes the “interconnection between people, animals, plants, and their shared environment” and brings together relevant stakeholders in human, animal, environmental health, and other sectors and disciplines [15, 16] — to guide current and future laboratory leaders in building, strengthening, and driving national laboratory systems.

### Model list of essential in vitro diagnostic devices (EDL)

Dr. Francis Moussy, the Team Lead of the Secretariat of the WHO Model List of Essential In Vitro Diagnostics (EDL) at WHO Headquarters, presented on behalf of the EDL Secretariat on the model list of essential IVD devices. The model list of essential IVDs [17] — presently in its third iteration — is an evidence-based document consisting of a registry of IVD test categories and recommendations for these tests, including information on test format, test purpose, sample types, and health system levels. The EDL aims to support IVD policy development to improve individuals' access to IVD testing and clinical laboratory services and includes general and disease-specific IVDs for both noncommunicable and infectious diseases.

Dr. Moussy proceeded to provide an overview of how the EDL is presented, detailing that there are two tiers — community settings and health care facilities without laboratories and health care facilities with clinical laboratories — which is presented alongside a “Do Not Recommend” section that includes tests categories that have been listed for discontinuation. The EDL is regularly updated though applications submitted by WHO Country Offices and other stakeholders, such as Member States, and overseen by the EDL Secretariat and recommendations provided by the Strategic Advisory Group of Experts. The fourth EDL is intended to be launched and published in January of 2023.

Further, Dr. Moussy outlined the nine steps for updating the EDL, which is a rigorous evidence-based process, the guiding principles for developing a national list of essential IVDs, discussed the committees and their respective functions, and noted four tools that can be used to support countries.

Lastly, Dr. Moussy concluded by providing key weblinks and the email address for the EDL Secretariat and encouraged participants to reach out by email with any questions or comments.

Dr. Coulibaly also indicated that countries were asked if they would be willing or interested to develop an EDL, and following this work, there will now be actions taken to host a workshop on how to develop an EDL.

### Primary health care (PHC) & laboratory and diagnostic services

This presentation was conducted by two different speakers. The synopsis for the presentation in French by Dr. Tarcisse L. Elongo is presented first, followed by the synopsis for the presentation in English by Dr. Gertrude Avortri.

Dr. Tarcisse L. Elongo, a Technical Officer in the Integrated Service Delivery/Primary Health Care Unit at the WHO AFRO, began by noting that a health system is defined as all activities whose primary purpose is to promote, restore, and/or maintain health, or people, institutions, and resources, arranged in accordance with established policies, to improve the health of the population they serve, while meeting people's legitimate expectations and protecting them from the costs of ill health through a variety of activities whose primary intent is to improve health. PHC supports UHC and the SDGs through improving access to efficient and equitable health services in a cost-effective manner. Its operational framework includes four strategic levers, which are: (i) political commitment and leadership, (ii) governance, (iii) financing and allocation/resources, and (iv) community and other stakeholder engagement. At the operational level, PHC supports: a model of care; strengthening the health workforce; physical infrastructure; drugs and other medical products; engagement with the private sector; procurement of services and payment models; digital technologies; quality, PHC-focused research; and monitoring and evaluation.

Further, Dr. Elongo provided references for in-depth information on International Health Regulations [18], One Health [19], and Integrated Disease Surveillance and Response [1].

Dr. Gertrude Avortri, Technical Officer of Integrated Service Delivery and PHC, began the presentation on PHC and laboratory and diagnostic services by defining PHC as “a whole-of-society approach to health that aims to ensure the highest possible level of health and well-being and their equitable distribution by focusing on people’s needs and preferences (as individuals, households, and communities) as early as possible along the continuum from health promotion and disease prevention to treatment, rehabilitation, and palliative care, and as close as feasible to people’s everyday environment” [2]. Dr. Avortri stressed that there are three interrelated components of the PHC approach to service provision, which are: (i) multisectoral policy and action, entailing addressing the broader determinants of health through evidence-informed policies and actions across all sectors; (ii) empowering people and communities, by advocating for policies that promote and protect health and well-being, as co-developers of health and social services, and as self-carers and caregivers; and (iii) PHC and essential public health functions as the core of integrated health services, through meeting people’s health needs through a continuum of services throughout the lifecourse.

In painting a picture of these interrelated parts and processes, Dr. Avortri noted that PHC is the approach, health systems are the means, and UHC and the health-related SDG targets are the goals. Thus, PHC can support UHC and the health-related SDGs. Operationally, the framework of PHC contains four strategic levers: (i) political commitment and leadership, (ii) governance and policy framework, (iii) funding and allocation of resources, and (iv) engagement of community and other stakeholders; and is supported by an additional ten operational levers [20].

Dr. Avortri further explained that PHC has implications for laboratory and diagnostic services, such as in providing essential health services (e.g., diagnostics and case management); screening, epidemiological surveillance, and preparing and responding to emergencies; and given relevance and engagement in multisectoral initiatives (e.g., in containing zoonotic diseases, conducting environmental surveillance).

To conclude, Dr. Avortri posed several questions to participants, including asking whether country departments in charge of laboratory and diagnostic services are well-positioned within their respective ministries of health.

### Antimicrobial resistance control and laboratory systems

Dr. Laetitia Gahimbare from the antimicrobial resistance (AMR) team at WHO AFRO presented at both meetings and began each presentation by pointing to historical images of penicillin and noting that we are facing the unfortunate reality that penicillin may no longer be able to be relied on. As a result, AMR is a global health priority and crisis that cannot be ignored.

Dr. Gahimbare noted that within the Global Action Plan for AMR, there are five strategic objectives: (i) improve awareness and understanding of AMR, (ii) strengthen knowledge through surveillance and research, (iii) reduce the incidence of infection through effective hygiene and infection prevention and control, (iv) optimize the use of antimicrobial medicines in human and animal health, and (v) ensure sustainable investment through research and development. Although all areas are important, laboratories play an important role in the second strategic objective. After the Global Action Plan for AMR was launched, Member States asked the WHO to put in place a surveillance system for which the Global AMR and Use Surveillance System (GLASS) was established. GLASS is the first global system to incorporate official national data from surveillance of AMR. It provides a standardized approach to the collection, analysis, and sharing of data, is a One Health model for AMR surveillance, and generates data to inform AMR burden estimates. Despite the initial focus of GLASS being bacterial infections in humans, it has evolved since its launch in 2015 to include routine data surveillance, focused surveillance, and surveys and studies. Within the WHO African Region, 33 countries have enrolled in GLASS and 28 countries are enrolled in the AFRO Microbiology Laboratory External Quality Assurance Program.

Dr. Gahimbare illustrated the roles laboratories play in AMR surveillance by pointing to various GLASS indicators relevant in country-level implementation (e.g., number of surveillance sites, designation of a national reference laboratory, antibiotic susceptibility testing, external quality assurance programmes), and noting the various functions national reference laboratories play in AMR surveillance, namely, reference; guidance and standardization; training; data collection and analysis; evaluation visits; and research. Dr. Gahimbare reviewed the resources available to countries as part of GLASS, including manuals, information technology tools, the help desk, webinars, country missions, training workshops, external quality assessment, and procurement.

At present, limitations to GLASS results include data completeness and representativeness, a need to strengthen laboratories, and a need for UHC. Ultimately, to improve the management of AMR across the laboratory, there is a need to strengthen laboratory capacities, namely through integration within programmes and laboratory capacity-building through a coherent strategy. Dr. Gahimbare concluded by providing reference materials for participants.

### Integrated laboratory systems contributions to disease control programmes

Dr. Jean de Dieu Iragena, a Laboratory Technical Officer, also presented at both meetings and began each presentation describing the contribution of integrated laboratory systems to disease control programmes by recalling various relevant laboratory resolutions. These include the Maputo Declaration of 2008 [21], the Yaoundé Resolution of 2008 [22], launch of the WHO-AFRO Kigali Stepwise Laboratory Accreditation programme in 2009 [23], and the 2010 Kampala Statement and subsequent launch of the African Society for Laboratory Medicine in 2011 [24]. Dr. Iragena highlighted these numerous actions to demonstrate that there is ongoing work in this domain.

With respect to relevant disease control vertical programme areas, laboratory systems are pertinent for four key programmes: (i) human immunodeficiency virus (HIV), tuberculosis, and hepatitis, (ii) tropical and vector-borne diseases, including malaria and neglected tropical diseases; (iii) noncommunicable diseases; and (iv) vaccine-preventable diseases. However, due to various disease programmes, diagnostic platforms for integrated diseases are highly uncoordinated (i.e., working in silos). This challenge is faced alongside numerous other challenges, such as around the slow uptake and insufficient utilization of available diagnostic technologies, intermittent supply and stock-out alongside equipment failures, a lack of formal structure in the diagnostic network that inhibits efficient functioning, issues with specimen referral systems, a lack of translation of diagnostic policies into practice within countries, a lack of national laboratory strategic and operational plans, a lack of sample referral systems to optimize the use of instruments, and a lack of clinician training creating a gap in understanding and inhibiting communication between laboratory technicians and clinicians.

Nevertheless, despite these challenges faced, there are opportunities presented following the COVID-19 pandemic, such as around the increased use of community health workers for tuberculosis, enhanced infection prevention and control, enhanced contact tracing, development of contingency plans, and other innovations and opportunities. However, there is a need to be strategic in considering opportunities to enhance efficiency. As an example, an increase of GeneXpert devices can be better capitalized on to test for various diseases at once (e.g., Ebola, HIV, SARS-CoV-2, tuberculosis). Dr. Iragena emphasized the importance of strong partnerships and networks, namely, the need for dialogue between programmes involving laboratory directorates.

## Thematic exchanges

Thematic exchanges were planned around six key areas, which are: (i) laboratory governance, (ii) laboratory policy and planning, (iii) regulation and legislation, (iv) partnerships, (v) management of laboratory technology, and (vi) national laboratory networks. The subsequent section details the experiences of sample countries around each theme.

### Laboratory governance

#### Guinea

Pr Mandiou Diakite, the Director of the National Laboratory Directorate at the Ministry of Health of Guinea, presented on Guinea’s experience. Pr Diakite explained that Guinea has a National Directorate of Laboratories whose mission is to develop, implement, and monitor government policy in the medical biology field. Guinea has an autonomous National Directorate of Laboratories, an organic framework validated by the civil service, and acts as a headquarters to contain policy texts, a master plan of medical biology, and normative documents. Laboratories, both public and private, participate in the external quality assurance programme. Further, projects exist to strengthen laboratories (e.g., Labogui, Redisse, Résaolab, Centers for Disease Control and Prevention, GLLP).

However, some shortcomings are still faced, including: insufficient and inadequate human, financial, and technical resources; failure to consider all aspects of diagnostics in pharmaceutical law; weak coordination of partners' interventions; insufficient mechanisms for financing and sustainable management of medical biology activities, as well as poor functioning of the laboratory network; non-respect of the attributions between the National Directorate of Laboratories and certain institutions involved in the laboratory; absence of a national quality assurance program; and weak involvement of management in the procurement process for the acquisition of laboratory equipment and consumables.

#### Sierra Leone

Ms. Victoria Katawera, the Team Lead for Laboratories at the Sierra Leone WHO Country Office, presented on behalf of the Ministry of Health and Sanitation, as the focal person was unavailable. Ms. Victoria Katawera began by providing a brief history of the public health laboratory system in Sierra Leone, noting that the National Laboratory Services was previously operating under the Directorate of Hospitals and Laboratory and has since moved under the Directorate of Laboratories, Diagnostics, and Blood Services, which was established in 2018.

Ms. Katawera discussed how a lack of integration of units and reporting lines led to a programmatic approach to laboratory service delivery, leading some laboratories to function in two distinct capacities: public health functions and clinical laboratories. However, the biggest challenge experienced was that these two laboratories were not speaking to each other. By operating in silos with their own meetings, it was difficult to foster a culture of shared communication. As a result, there were some organizational changes undertaken by the Ministry of Health and Sanitation to revitalize laboratory, diagnostic, and blood services in Sierra Leone through changing reporting lines. Namely, a phased approach was taken, primarily so that relationships with stakeholders could be better managed.

Although progress has been made, this work is ongoing. The aim is to have public health laboratories integrated with clinical laboratories as a national laboratory service, returning to how it was previously organized. Ms. Katawera emphasized that collaborations and linkages with stakeholders need to be continued and that there must be coordinated management at this level. Because most of the regional public health laboratories are situated in clinical laboratories, without coordination mechanisms, difficulties are faced. Therefore, one step taken was to appoint a director who could enact changes at the directorate level.

Ms. Katawera further discussed some of the drivers of this process, namely the support of stakeholders such as the laboratory focal person(s) understanding and appreciating the need for integration, Ministry of Health and Sanitation leadership support, and partner support; and other key drivers like proper planning, soft skills, understanding stakeholders’ interests and managing these, and diplomacy. However, challenges faced included resistance and human resources, both in terms of quantity and capacity.

Ms. Katawera noted that the WHO’s role in this case was to speak to partners and frequently remind stakeholders that this is the best way to proceed. Ms. Katawera further concluded that because there is support and commitment, progress is being made in observable ways.

### Laboratory policy and planning

#### Togo

Dr. Fiali A. Lack, Medical Doctor and Clinical Laboratory Specialist at the Lomé teaching hospital, who represented the Togo Director of Laboratory at the Ministry of Health, shared Togo’s experiences. Dr. Lack explained that the Strategic Plan for the Development of Medical Biology in Togo builds on the National Health Development Plan to improve clinical care, public health programmes, and the health of all Togolese communities and outlines a series of strategic actions in response to the challenges of the sector and covers logistical, financial, technical, and quality aspects.

The strategic objectives for 2018–2022 are to: reorganize and revitalize the coordination of medical biology laboratory activities; provide the knowledge, skills, and tools necessary to improve the quality of laboratory services by motivating and empowering laboratory professionals; define standard laboratory test packages for each level of care in the health system; design models of laboratory plans; and better supervise the acquisition of equipment and reagents.

Further, Togo has established a national policy (2018–2027) and a strategic plan (2018–2022) that includes specific objectives and activities. However, there are some shortcomings, particularly: the absence of a validated legislative and regulatory framework, an insufficient integration of activities across different laboratory networks, challenges implementing the strategic plan given insufficient financial support, and the quality management system.

#### Zimbabwe

Drs. Raiva Simbi, the Director of Laboratory Services at the Ministry of Health, and Muchaneta Mugabe, from the Zimbabwe WHO Country Office, presented on Zimbabwe’s laboratory policy and planning landscape. They began their presentation by explaining that the country has a standalone laboratory policy document. However, the mission and vision are derived from the National Health Strategy, which is also derived from the ministerial integrated performance agreement.

Drs. Simbi and Mugabe also noted that Zimbabwe’s strategic laboratory plan, developed alongside development partners who continue to review the strategic plan, was adopted by the Ministry of Health’s Permanent Secretary for Health. The strategic plan has been reviewed to cover a five-year period and includes all laboratory activities. However, they observed that select areas may require separate strategies that can be attached as addendums (e.g., point-of-care tuberculosis and HIV, biosecurity and biosafety strategies).

Laboratory activities undertaken in Zimbabwe, both old and new, are strategically sought to align with the strategic plan. Although the plan is partially funded — with a commitment from both partners and the government — Drs. Simbi and Mugabe noted that funding is never enough.

Following Drs. Simbi and Mugabes’ presentation, Dr. Coulibaly made the astute observation that in some countries, the strategic plan acts as merely a document and is not being utilized in practice. In other words, although it has been developed, not all partners are aware of its existence. Consequently, it is important to ensure that the strategic plan plays its intended role — to orient laboratory activities and development. Therefore, securing commitment from partners and having a strategic plan that can tangibly orient everyone is essential.

### Regulation and legislation

#### Senegal

Dr. Aminata Diop from the Laboratory Directorate in Senegal’s Ministry of Health presented on laboratory systems in the country, highlighting the status of laboratory regulations and legislation. Dr. Diop noted that the legislative and regulatory framework in Senegal consists of a series of laws and decrees (listed in Table 1). However, there are some shortcomings in Senegal around: the post-inspection follow-up of laboratories, particularly when seeking to factor in the development of illicit or illegal medical biology practices; absence of a harmonized and integrated system for transporting biological samples (e.g., draft decree); sending samples abroad; and delays in registering laboratory inputs.

**Table 1:** List of relevant laws and decrees in Senegal

• Law n°2009–11 of January 23, 2009, relating to medical analysis laboratories
• Decree n°2009–364 of April 20, 2009 implementing law n°2009–11 of January 23, 2009 relating to medical analysis laboratories
• Decree n°2009–365 of April 20, 2009, fixing the conditions of registration and distribution of reagents used in medical biology laboratories
• Decree n°2009–366 of April 20, 2009, on the organization and functioning of the national commission of medical biology
• Decree No. 2012–543 of May 24, 2012, creating the Directorate of Laboratories
• Order No. 00275 MSPM/CAB/BL of February 3, 2005, establishing a National Network of Laboratories (RNL)
• Order No. 6527 MSPHP/CAB/RNL of June 23, 2009, on the organization of the RNL
• Order No. 001230MSP/DPL/LABM of February 2, 2011, establishing the conditions for registration and marketing of medical biology analysis reagents
• Order No. 001283 MSP/DPL/LABM of February 3, 2011, establishing the conditions for approval of medical analysis reagent distribution companies
• Order No. 003516 of March 11, 2013 on the designation and organization of training and quality assurance laboratories
• Order No. 011023 MSAS/DGS/DL of July 04, 2014, mapping the possibilities of creating private medical biology laboratories
• Order No. 14897 MSAS/DGS/DL of September 19, 2014, on the creation of secondary sampling centers external to private medical biology laboratories
• Order No. 10947 MSAS/DGS/DL of March 29, 2015, on the approval of companies responsible for importing and distributing laboratory reagents and consumables in Senegal
• Order No. 13128 MSAS/DGS/DL of July 2, 2015, establishing and setting the rules of organization and operation of the National Public Health Laboratory (LNSP)
• Order No. 00636 MSAS/DGS/DL of January 20, 2016, appointing the Director of the LNSP
• Order No. 13886 MSAS/DGS/DL of August 07, 2017 on the adoption of the ISO 15189 standard in the LBM
• Order No. 001970 of February 12, 2018 on the standard of facilities, equipment and personnel for the LBMs of public health structures

#### Ghana

Dr. Awininbuno Ignatius from the Ministry of Health began the presentation on Ghana’s experience with regulation and legislation by providing a bit of background information on the diagnostic and laboratory regulatory system in Ghana, explaining that it is a four-tiered national system. Dr. Ignatius explained that over many years, the implementation of quality management systems has drastically improved, resulting in three public sector laboratories being accredited to ISO 15189 and two public health laboratories certified to ISO 9001. Dr. Ignatius further noted that more laboratories are moving from paper-based data management to electronic systems, which is contributing to improved service, data availability and quality, and laboratory usage.

Dr. Ignatius explained that in Ghana, there are two branches of laboratories: clinical and public health. With respect to the former, Ghana has 500 clinical laboratories, including 16 regional hospital laboratories and 216 district laboratories. And regarding the latter, there are five public health laboratories that are responsible for disease surveillance, outbreak investigations, control, prevention, training, monitoring, and education within the laboratory network.

In addition to the clinical and public health laboratories, Ghana also has: five self-managed teaching hospital laboratories that are overseen by the Ministry of Health, three academic research laboratories, 344 faith-based organization and 34 religious-organized laboratories, quasi government laboratories, approximately 400 private laboratories, and 14 veterinary laboratories.

Dr. Ignatius provided a brief overview of relevant regulations, legislation, and authorities that pertain to laboratories (listed in Table 2). However, despite the numerous regulations, pieces of legislation, and authorities in place, Dr. Ignatius noted that laboratory systems in the country face select challenges. Notably, most regulations lack legislation; there is ineffective regulation of the Allied Health professionals’ Council, rendering it ineffective; lack of a regulatory body that can adequately ensure qualified and competent professionals are licensed to practice; the inspectorates’ unit is weak and often lacks expertise in laboratory services; and occasionally, laboratory professionals are not part of inspectorate teams.

**Table 2:** List of relevant regulations, legislation, and authorities in Ghana

• The Health Professions Regulatory Bodies Act, 2013 (ACT 857) Part One Allied Health Profession Council
◦ With the passage of this act, medical laboratory practice became a regulated field of practice and training alongside 17 other professionals in Ghana
• Part one of Health Facilities Institutions Act 829:2011, established by the Health Facilities Regulatory Authority, provides requirements or standards for laboratories to operate and continuously do so via licensing
• The Ghana Standards Authority provides standards (GS/ISO 15189:2017) and metrological services to laboratories
• The Ghana National Accreditation Service grants accreditation to laboratories in accordance with relevant international standards
◦ However, it currently lacks a parliamentary Act to back it and is poorly resourced
• The Food and Drugs Authority ensures that diagnostic devices go through a verification process before granting marketing authorization
• The Ghana Health Service and Teaching Hospital Act 525:1996
• The Ghana Technical and Education Commission collaborates with the Allied Health Profession Council in approving training curriculums (e.g., Doctor of Medical of Laboratory Science Program)
• The Ghana Association of Medical Laboratory Scientists provides professional self- regulation
• The Public Health Act

### Partnerships

#### The Democratic Republic of the Congo

Mr. Malaba Munyandji Cléophas, the Director of Laboratory Services at the Ministry of Health in the Democratic Republic of the Congo, spoke about partnerships. Mr. Cléophas expressed that it is essential to draw on partnerships to improve the quality of service delivery and to accelerate project implementation. In a partnership, entrusting a single private partner with these steps can substantially shorten the time required for completion, promote better risk management, and increase the productivity of public administration.

In the Constitution of the Democratic Republic of the Congo — namely article 122, points 3, 8, and 11 — the legal provisions applicable to the contracts of public–private partnerships are outlined. In this legal provision, the rules concerning "public finance, trade, the system of ownership of civil and commercial rights and obligations, as well as loans and financial commitments of the state” are detailed.

However, the Democratic Republic of the Congo faces challenges with partnerships in the health sector due to: insufficient coordination mechanisms (e.g., consultation framework, inequitable distribution of partners) and the regulatory role of the state; muddied roles and obligations of each partner (e.g., conflict of interest between framework agreements and service delivery contracts); inability to negotiate due to an imbalance of power; insufficient transparency; and weak involvement of beneficiaries and field actors.

It is therefore recommended to: ensure that public–private partnership contracts are long-term agreements while recognizing that it is difficult to correctly anticipate problems that may arise in the distant future; adopt regulations that can provide legal certainty for investors and clear procedures for resolving disputes, should it be necessary to renegotiate contracts; correctly assess costs and benefits of each partnership request with the private sector to make the investment more profitable, factoring their lifespan and the complexity of this type of financing; secure expertise to negotiate and effectively manage the execution of contracts, given that public infrastructure investment projects with partnerships are complex; and have institutional structures and a legal framework to protect contractual and property rights.

#### Nigeria

Dr. Kingsley Odiabara, the Director of Medical Laboratory Services at the Federal Ministry of Health in Nigeria, presented on partnerships. Dr. Odiabara began the presentation with a brief overview of the governance structure in Nigeria and explained that the Medical Laboratory Services Division is situated under the Department of Hospital Services and is led by a director who is a medical laboratory scientist. Dr. Odiabara explained that there are five regulatory bodies in Nigeria: the Medical Laboratory Science Council of Nigeria, the Medical and Dental Council of Nigeria, the National Agency for Food and Drug Administration and Control, the National Biosafety Management Agency, and the Standard Organization of Nigeria, all of which are responsible for implementing the Nigeria National Medical Laboratory Services Policy 2021–2025, which was approved by the Honourable Minister of Health. Furthermore, the supplemental strategic plan has been validated by stakeholders and is awaiting approval from the Minister.

Dr. Odiabara further explained that the Medical Laboratory Services Division is responsible for coordinating the development and implementation of policies that affect Medical Laboratory Services in Nigeria. In doing so, the division liaises with laboratories in staff clinics and tertiary health institutions, relevant regulatory bodies, international organizations, and agencies and programmes on laboratory networks. The division also collaborates with the Department of Hospital Services to oversee the activities of laboratories in the Federal Health Institutions and functions as secretariat to the National Laboratory Technical Working Group (NLTWG). With respect to the latter, the NLTWG was inaugurated by the Honourable Minister of Health in January of 2017. The NLTWG conducts advisory, oversight, and implementation support, and has seven sub-groups covering 21 thematic areas within the Nigerian National Medical Laboratory Service Policy and Strategic Plan. Dr. Odiabara also discussed how Nigeria has several disease-specific laboratory networks, such as the WHO polio network.

With respect to partnerships, Nigeria has had many innovations and milestones. For instance, establishing the National Integrated Specimen Referral Network policy, which was approved by the Honourable Minister of Health; effective sample referral for HIV/AIDS and tuberculosis; and progress made on developing an electronic national laboratory information management system. Looking ahead, Dr. Odiabara noted some of the medium-term priorities for laboratory strengthening, which include upgrading the Medical Laboratory Services Division to a full department level at the Federal Ministry of Health, implementing and expanding support for laboratory quality management system implementation, and developing an effective monitoring and evaluation framework for laboratory interventions in the country. However, Nigeria has also faced challenges with laboratory strengthening, including parallel and fragmented implementation by disease programmes, the lack of effective coordination across partners’ activities, and the lack of a laboratory map within the country. Thus, Nigeria has requested the support of WHO AFRO to assist with: funding to organize partner coordination quarterly, technical and logistical support for a country-wide laboratory assessment and mapping, capacity-building for confirmation of cerebrospinal meningitis, and funding support for a conference of national and sub-national laboratory directors.

### Management of laboratory technology

#### Gabon

Dr. Armel Mintsa Ndong, the Director of the National Public Health Laboratory at the Ministry of Health in Gabon, shared Gabon’s experiences managing laboratory technologies. Dr. Ndong explained that the National Public Health Laboratory is a health structure with a national vocation and technical and management autonomy. It was created in January 1960 and its mission is focused on executing and controlling government policy in clinical biology, recycling, practical training of staff, and clinical biology research. It ensures the budget allocation of the government through technical and financial partners (e.g., WHO, Africa Centres for Disease Control and Prevention, the African Society for Laboratory Medicine, the European Union).

However, Gabon’s management of laboratory technology faces challenges, such as: lacking a specific manual for equipment management; having no laboratory-specific national medical equipment maintenance service (however, there is a biomedical maintenance unit at the National Public Health Laboratory); inefficient service despite it being effective; no written texts governing the donation of equipment, but rather prioritizing the needs of donors; and the need to harmonize equipment to facilitate maintenance and quality control.

Therefore, it is recommended to: develop a national equipment management plan that integrates biomedical maintenance; identify a focal point for biomedical maintenance in all laboratories; ensure that a maintenance contract is signed when each piece of equipment is acquired; and encourage the acquisition of relay groups for each laboratory.

#### Zambia

Dr. Aaron Lunda Shibemba, the National Coordinator of Pathology and Laboratory Services in Zambia, began the presentation with a bit of background information on the vision and mission of the 2018–2022 National Biomedical Laboratory Strategic Plan. Dr. Shibemba noted that the vision is to have a functional and sustainable laboratory service for all Zambians and the mission is to provide Zambians with high-quality, accurate, timely, cost-effective, and appropriate laboratory services at all levels of care and as close to communities as possible. Dr. Shibemba then proceeded to discuss the core functions of the Ministry of Health laboratory unit, which include laboratory management and coordination, equipment procurement/placement and distribution, laboratory supply chain and coordination, clinical diagnosis, disease prevention and control, surveillance, reference and specialized testing, quality management systems, capacity-building for laboratory testing and personnel, and laboratory accreditation.

Further, Dr. Shibemba discussed the laboratory network in Zambia, explaining there are 534 functional laboratories that are geographically dispersed across the country and include: 25 molecular testing, 108 early infant diagnosis point-of-care; 33 viral load point-of-care, and 327 tuberculosis diagnosis using GeneXpert laboratories. 

In terms of laboratory equipment, Zambia has historically used an equipment “outright purchase” model. However, challenges with this model were identified, such as: the lack of equipment maintenance contracts, long equipment downtime, frequent service interruptions, obsolete equipment with no clear replacement plans in laboratories, and sub-optimal service provision. Given these issues, the equipment “placement” model was adopted in 2018. Originally with funding from United States President's Emergency Plan for AIDS Relief funding (PEPFAR) and now from the Ministry of Health — although identified as not being sustainable — has led to the successful integration of all HIV/AIDS viral load and early infant diagnosis machines, including GeneXpert, through service-level agreements. Dr. Shibemba explained that the Ministry of Health has had meetings with suppliers of other equipment, such as hematology and chemistry, but the suppliers are slow to migrate and sign memorandums of understanding because of unassured or unsustained procurement of reagents and consumables. However, through the USAID global health supply chain program, Zambia was able to achieve better cost pricing by leveraging all PEPFAR volume, leading to cost savings of $2.2 million by the end of 2021.

Dr. Shibemba proceeded to explain that with the new reporting process approach, the Ministry of Health reviews vendor performance quarterly against key performance indicators, leading to improved transparency across three major buckets of performance. Further, the Ministry of Health published the Guidelines for the Approval of Laboratory Equipment and Commodities in June of 2021 and associated new equipment functionality module, which allow for monitoring progress and mitigating challenges. Thus, facilities are now able to report test numbers in real-time, allowing for the validation of reported facility consumption data.

In concluding, Dr. Shibemba pointed to the key challenges of old and obsolete equipment; frequent equipment breakdowns, especially for haematology and chemistry; long equipment downtime; and reagent and consumable stockouts; leading to the key message that if Africa wants to ensure sustainability, the continent must manufacture their own equipment and reagents.

### National laboratory networks

#### Burkina Faso

Dr. Zakariya Yabre, the Director General of Access to Health Products in the Ministry of Health in Burkina Faso, presented on the country’s national laboratory networks. Dr. Yabre first provided historical context by explaining that before 2014, Burkina Faso’s reference laboratories and the national network of laboratories existed informally without a regulatory framework and with unclear missions. This situation was detrimental to the organization of infectious disease surveillance and diagnostic expertise.

Notably, the national laboratory network was comprised of several sub-networks, including the tuberculosis laboratory network, meningitis laboratory network, and HIV laboratory network, with the network operations being the responsibility of respective health programs and not involving laboratory management.

Thanks to the institution of national reference laboratories, the network was formalized in 2014 through a ministerial order and is built on the pyramid model of the health system. The network includes all public and private laboratories and is classified according to the health pyramid: laboratories at the peripheral level, intermediate, or regional level; laboratories at the national level; and the national reference laboratories.

However, Burkina Faso also faces challenges with respect to the national laboratory network around: low laboratory coverage in semi-urban and rural areas despite the project to transform first level health facilities in rural communes into medical centers (2d level) — approximately 60 first level health facilities transformed out of 256 since 2013; the expiration of the mandate of seven national reference laboratories in 2019, which have not yet been renewed; insufficient response to health emergencies due to the continuous unavailability of inputs and insufficient human resources both in terms of quantity and quality; the fact that the central reference laboratory that should coordinate the activities of the national reference laboratories is not yet operational; weaknesses in the financing of the national reference laboratories to implement laboratory and medical biology policies; insufficient laboratory information systems; and vertical interventions by partners, given that vertical approaches have occasionally led to the fragmentation of laboratory services despite also improving disease-specific responses.

#### Rwanda

Dr. Isabelle Mukagatare and Mr. Robert Rutayisire, the Head and Division Manager of the Biomedical Sciences Department at the Rwanda Ministry of Health, respectively, jointly presented on the national laboratory network in Rwanda. Their presentation began with Dr. Mukagatare clarifying that the Rwanda Biomedical Centre is the policy implementing agency of the Ministry of Health. Mr. Rutayisire then noted its mission, which is to provide accessible quality laboratory services and strengthen the national diagnostic network through leadership and expert guidance to reduce the burden of disease in Rwanda and in the Region; and vision, which is to become a coordinated center of excellence for medical laboratory diagnosis by providing quality laboratory services to ensure healthy people for a wealthy nation.

Next, Mr. Rutayisire presented an overview of the structure and services in the national laboratory network, explaining that this includes six essential units: laboratory services coordination and quality assurance unit, the microbiology unit, the immunovirology unit, the molecular and genomics unit, the clinical pathology unit, and the medical entomology unit. Mr. Rutayisire further elaborated on the description of the Rwanda laboratory network, noting that it includes four levels: (i) level one, which includes the public health centres (*n* = 561), health posts (*n* = 1094), and private clinics (*n* = 318); (ii) level two, which are the district hospitals (*n* = 41); (iii) level three, which are the provincial hospitals (*n* = 4); and (iv) level four, which are the referral hospitals (*n* = 8) — with the national reference laboratory overseeing the work of all four levels. Although level one only performs routine tests, levels two, three, and four perform Nucleic acid, GeneXpert, and routine tests, and the national reference laboratory conducts advanced and routine testing, provides oversight of the laboratory network, and ensures the implementation of quality management systems at all levels.

Overall, the national reference laboratory seeks to align its priorities with the vision of the government of Rwanda to transform the system into a world-class medical laboratory network that can deliver quality, reliable, affordable, and accessible health services to all Rwandans. It was noted that the service delivery performance of the laboratory network is strong and consistent with short turn-around times between sample collection and results, an effective linkage to care, and high-quality laboratory services and clinical management for patients. Further, all sites can reliably provide all diagnostic tests outlined in the Ministry of Health benefit package at each tier of the health system, without interruption or delay, through referral when necessary. Additionally, financial resources in the diagnostics network are allocated strategically, as they are efficiently managed, transparent and visible to leadership, and lead to value created for each Rwandan franc spent.

Mr. Rutayisire noted that the laboratory network has been in the process of optimization since 2019 and the increased network capacity is expected to continue to increase by 2.4 times from 2019 to provide capacity to cover the full HIV/AIDS viral load demand. It was noted that for tuberculosis, the viral load network, and for early infant diagnosis, there are 548 collection sites and specimens are referred to the closest testing sites. Further, the decentralization of sample referral vehicles — now with seven trucks covering 14 hubs in the country, resulting in an estimated 250,000 km travelled per year — is expected to significantly reduce distances travelled — leading to six sites covered by 15 trucks, resulting in an estimated reduction of distance travelled by 140,000 km per year.

Mr. Rutayisire explained that at present, the key priorities for the national reference laboratory are to improve: access to quality services, by seeking to establish a world-class laboratory and five regional national reference laboratories across the country; quality management systems, through enrolling and supporting the eight laboratories in the accreditation process, strengthening mentorship and supervision in laboratories, and establishing a local accreditation body; infrastructure and equipment, through further simplifying testing methods by introducing point-of-care and decentralizing and task-shifting to increase access to quality and timely diagnostic services; network optimization, by planning exercises to inform the future placement of equipment and decentralizing laboratory services; data systems, by automating the release of results to patients, ensuring connectivity of all diagnostic equipment and laboratory information systems, and shifting from disease-centred data systems to integrated laboratory management information systems; and human resources, by strengthening capacity or quality, increasing quantity, and strengthening the e-learning platform for continued education.

However, in concluding, Mr. Rutayisire also noted two main challenges Rwanda is facing, which are the need to achieve an optimal level of inter-operability across various public health data systems and the limited number of staff at the national level. Mr. Rutayisire then invited participants to consider solutions to these challenges.

## Conclusion

With the presentations from various WHO Technical Officers and experiences shared by representatives from countries in the Region, not only were laboratory leaders able to learn from best practices and anticipate challenges for their own respective countries, but participants had the opportunity to engage in rich plenary discussions and question and answer periods. This engaged dialogue allowed participants to better understand the situation, actions, and solutions in countries, and ultimately, to draw cross-country comparisons. Further, outlined challenges and areas for improvement noted in presentations can guide collective action and inform WHO staff where additional support can be provided, whether at the Country Office, Regional Office, or Headquarters level. Ultimately, beyond the scope of seeking to address outlined challenges, these meetings served to create a platform for laboratory leaders to foster connections that will allow them to connect over the course of their careers. Further, it provided a reminder that this meeting signals the beginning of many fruitful collaborations and opportunities in the Region in laboratory and diagnostic services. It is anticipated that similar meetings will be held in future years to build on the momentum fostered to move the needle for enhanced PHC and striving for UHC, and ultimately, working towards reaching health and related SDG targets.

## Supplementary Information

Supplementary Material 1. List of participants.

## Data Availability

Not applicable.

## Abbreviations

AFRO: WHO Regional Office for Africa
AMR: Antimicrobial resistance
CRP: Collaborative registration process
EDL: Essential in vitro diagnostic devices
GBT + MD: WHO Global Benchmarking Tool + Medical Devices
GLASS: Global AMR and Use Surveillance System
GLLP: The Global Laboratory Leadership Programme
HIV: Human immunodeficiency virus
IVD: In vitro diagnostic
MDs: Medical devices
NLTWG: National Laboratory Technical Working Group
NRAs: National regulatory authorities
PEPFAR: United States President's Emergency Plan for AIDS Relief funding
PHC: Primary health care
PQ: Prequalification
SDGs: Sustainable Development Goals
UHC: Universal Health Coverage
WHO: World Health Organization
