# Supplementary material for: World Health Organization African Region national heads of units of diagnostics and laboratory services meetings proceedings
Source: BMC Proc. 2024 Nov 28;18(Suppl 17):26. doi: 10.1186/s12919-024-00305-1 (PMC11603677; doi:10.1186/s12919-024-00305-1)
Supplement: Supplementary file 1 — Supplementary Material 1. List of participants. [file 12919_2024_305_MOESM1_ESM.docx]

# Supplementary file

**List of participants**

| **COUNTRY** | **PARTICIPANT** | **PROFILE/ORGANIZATION** |
| --- | --- | --- |
| ANGOLA | Ms. Carvalho Marcia | Director public health diagnostics,_**MoH** |
| BENIN | Dr. ONIFADE Al Fattah | NPO/EDM/OMS/BENIN |
| BENIN | Dr. ZOHOUN Alban Gildas Comlan | Directeur des Exploitations Diagnostiques_ MINSANTE |
| BOTSWANA | Mr. Mbulawa Mpaphi | Director Health Lab services,_**MoH** |
| BOTSWANA | Dr. Madidmalo Tebogo | NPO- HIV_**WHO** |
| BURKINA FASO | Dr. GAMPINI Sandrine Estelle | EDM/HSS/OMS/BURKINA FASO |
| BURKINA FASO | Dr. YABRE Zabariya | Directeur Général de l'accès aux produits de santé_ MINSANTE |
| BURUNDI | Dr. NIYOMWUNGERE Alexis | Technical Officer (lab specialist)_OMS/BURUNDI |
| BURUNDI | Dr. NONERA Jean Marie | Directeur des Laboratoire de Biologie Médicale - MINSANTE |
| CABO VERDE | Dr. VEIGA Maria Elisa Mendes Da | Coordinatrice du réseau de laboratoires -MINSANTE |
| CAMEROON | Dr. BESONG Samuel | NPO/EDM/OMS/CAMEROUN |
| CAMEROON | Dr. MBWE MPOH Maurice | Directeur des Laboratoires _MINSANTE |
| CANADA | Dr. Michelle Amri | Consultant,_**WHO/AFRO** |
| CENTRAL AFRICAN REPUBLIC | Dr. KONGBELET ZINGAS NEE MBERYO Y. F. S | NPO Surveillance EPR/OMS/CENTRAFRIQUE |
| CHAD | Dr. Oumar Ouchar Mahamat | MINSANTE |
| COMOROS | Mme SAID ALLAOUI Zaharia | Directrice des Etablissements de soins publics et privés _MINSANTE |
| CONGO | Dr. BAKALA Dieudonné | EDM/OMS/CONGO |
| CONGO | Dr. AKIANA Jean | MINSANTE |
| CONGO | Dr. Sunday Kisoma | Consultant,_**WHO/HQ** |
| CONGO | Bertha Kembabazi | IT systems,_**WHO/AFRO** |
| CONGO | Itoua Marcelline | Administrative Assistant /WHO/AFRO |
| CONGO | Mavoungoud Mireille | Administrative Assistant /WHO/AFRO |
| CONGO | Dr. Gahimbare Laetitia | Technical officer AMR_**WHO/AFRO** |
| CONGO | Dr. Margaret Alia Samson Paul | Technical officer,_**WHO/AFRO** |
| CONGO | Dr. IRAGENA Jean de Dieu | Laboratory Technical Officer,_**WHO/AFRO** |
| CONGO | Dr. Sheick Oumar Coulibaly | Team Lead, Diagnostic and Laboratory Services Unit/ARD Cluster, WHO/AFRO |
| CONGO | Lorna Maria Aine | Data manager |
| CONGO | Dr. Sougou Aissatou Sarassa | Technical Officer (essential drugs and medicine) |
| CONGO | Dr. Tarcisse Elongo | OMS/AFRO |
| DRC | Dr. Kindambu Nyota Marie-France | Conseiller Pharmaceutique _OMS/RDC |
| DRC | Mr. Malaba Munyandji Cléophas | MINSANTE |
| DRC | Jérémie Muwonga Masidi | PNLS |
| ESWATINI | Ms. Sindiswe Susan Dlamini | Chief laboratory technologist,_**MoH** |
| ESWATINI | Dr. Mekdim Ayana | HSS Team Lead_**WHO** |
| FRANCE | Dr. Sebastian Cognat | Chef d'unité_ OMS/France |
| FRANCE | Dr. Virginie Dolmazon | Technical Officer _OMS/FRANCE |
| FRANCE | Dr. Lisa Stevens | Technical Officer_**WHO/HQ** |
| FRANCE | Dr. Céline Barnadas | Technical Officer_**WHO/HQ** |
| GABON | Dr. INOUA Aboubacar | Cluster Head/WCO/GABON |
| GABON | Dr. MINTSA NDONG Armel | Directeur Général _MINSANTE |
| GHANA | Dr. Awininbuno Ignatius | **MoH** |
| GHANA | Dr. Ackon Angela Ama | NPO-EDM_**WHO** |
| GUINEE | Dr. KOLIE Cécé Vieux | NPO/EDM_OMS/GUINEE |
| GUINEE | PR DIAKITE Mandiou | Directeur National des Laboratoires_MINSANTE |
| KENYA | Dr. Peter Borus Kimutai | Scientist_**WHO** |
| LIBERIA | Mr. Fahn Taweh Momo | Director Public Health Diagnosis and Laboratory_**MoH** |
| LIBERIA | Mrs. Mulbah Gertrude Jeh | Lab Technical Officer_**WHO** |
| MADAGASCAR | Dr. RAZAFINDRABE Falihery | Conseiller Pharmaceutique OMS/MADAGASCAR |
| MADAGASCAR | Dr. RAVAOARIMANANA Maxime | Directeur de la pharmacie, des laboratoires_MINSANTE |
| MALAWI | Mr. Joseph Bitilinyu Bangoh | Deputy director -national laboratory_**MoH** |
| MALAWI | Nyasulu Ishmael | NPO- HIV/TB/VN/EDM_**WHO** |
| MOZAMBIQUE | Dr. Felix Pedro Pinto | Head of department, mean auxillary for diagnostics (lab clinic, pathology, imaging and radio therapy,_**MoH** |
| MOZAMBIQUE | Dr. Sinesia Jose Sitao | Infectious hazard management and response officer_**WHO** |
| NIGER | Dr. SALISSOU ZAKARY Adamou | Team lead LABO_OMS/NIGER |
| NIGER | Dr. MOUMOUNI SAMBO Halimatou | Directrice des laboratoires de santé,_MINSANTE |
| NIGERIA | Dr. Kingsley Odiabara | Director medical laboratory services_**MoH** |
| NIGERIA | Dr. Ekanem Blessing | NPO Laboratory,_**WHO** |
| RWANDA | Dr. Isabelle Mukagatare | Head of Biomedical sciences department,_Ministry of Health, Rwanda |
| RWANDA | Stella Tuyisenge | WHO Country Office, Rwanda |
| RWANDA | Robert Rutayisire | Division manager- biomedical sciences,_ Ministry of Health, Rwanda |
| RWANDA | Frederique Geron | WHO |
| RWANDA | Lyndah Makayoto | WHE TEAM LEAD_ WHO Country Office, Rwanda |
| RWANDA | Habarurema Gaspaw | Ministry of Health, Rwanda |
| RWANDA | Cyitatire Ingrip | Ministry of Health, Rwanda |
| RWANDA | Kamarebe Noelle | Ministry of Health, Rwanda |
| RWANDA | Imaniriho Gabriel | Ministry of Health, Rwanda |
| RWANDA | Dr. Thierry Muvunyi | Consultant pathologist,_ **Ministry of Health, Rwanda** |
| SENEGAL | Dr. Jeff Maotela Kabinda | Health system coordinator/OMS/SENEGAL |
| SENEGAL | Dr. Aminata DIOP | MINSANTE |
| SIERRA LEONE | Ms. Victoria Katawera | Team Lead – Laboratory,_**WHO** |
| TOGO | OUEDRAOGO, Kiswendsida Romain H. | Coordinator, Health systems OMS/TOGO |
| TOGO | Lack A. Fiali | Médecin biologiste laboratoires CHU-SO_MINSANTE |
| TOGO | Sadji Adodo Yao | Chef de laboratoires à l'INH/MINSANTE/TOGO |
| TOGO | Issa Zoulkarneiri | Chef service épidémiologie et gestion des données_MINSANTE |
| UGANDA | Dr. Solome Okware | NPO Surveillance,_**WHO** |
| UGANDA | Dr. Susan Ndidde Nabadda | Commissioner national health laboratory & diagnostic services,**_MoH** |
| UGANDA | Martha Tusabe | Technical Officer -Bioengineering_**WHO/AFRO** |
| ZAMBIA | Dr. Aaron Lunda Shibemba | National coordinator pathology and lab services,_**MoH** |
| ZAMBIA | Dr. Shikanga Otipo | Epidemiologist,_**WHO** |
| ZIMBABWE | Dr. Raiva Simbi | Director laboratory services,_**MoH** |
| ZIMBABWE | Dr. Muchaneta Mugabe | WHE-EPR_**WHO** |
| ZIMBABWE | Dr. Edith A Annan | Technical Officer- Essential Drugs and Medicines, _**WHO/AFRO/IST/ESA** |
| ZIMBABWE | Dr. Gertrude Avortri | Technical Officer - service delivery systems, **WHO/AFRO/IST/ESA** |
